# Molecular landscape and prognostic impact of *FLT3*-ITD insertion site in acute myeloid leukemia: RATIFY study results

**DOI:** 10.1038/s41375-021-01323-0

**Published:** 2021-07-28

**Authors:** Frank G. Rücker, Ling Du, Tamara J. Luck, Axel Benner, Julia Krzykalla, Insa Gathmann, Maria Teresa Voso, Sergio Amadori, Thomas W. Prior, Joseph M. Brandwein, Frederick R. Appelbaum, Bruno C. Medeiros, Martin S. Tallman, Lynn Savoie, Jorge Sierra, Celine Pallaud, Miguel A. Sanz, Joop H. Jansen, Dietger Niederwieser, Thomas Fischer, Gerhard Ehninger, Michael Heuser, Arnold Ganser, Lars Bullinger, Richard A. Larson, Clara D. Bloomfield, Richard M. Stone, Hartmut Döhner, Christian Thiede, Konstanze Döhner

**Affiliations:** 1 Department of Internal Medicine III, University Hospital of Ulm, Ulm, Germany.; 2 Novartis Pharmaceuticals, Cambridge, MA, USA.; 3 Department of Hematology, Oncology and Tumor Immunology, Charité University, Berlin, Germany.; 4 Division of Biostatistics, German Cancer Research Center, Heidelberg, Germany.; 5 Novartis Pharmaceuticals, Basel, Switzerland.; 6 Department of Biomedicine and Prevention, Università di Roma “Tor Vergata”, Rome, Italy.; 7 The Ohio State University Comprehensive Cancer Center, Columbus, OH, USA.; 8 Department of Medicine, University of Alberta, Edmonton, AB, Canada.; 9 Clinical Research Division, Fred Hutchinson Cancer Research Center, Seattle, WA, USA.; 10 Division of Hematology, Stanford Comprehensive Cancer Center, Stanford University, Stanford, CA, USA.; 11 Leukemia Service, Department of Medicine, Memorial Sloan Kettering Cancer Center and Weill Cornell Medical College, New York, NY, USA.; 12 University of Calgary, Calgary, AB, Canada.; 13 Hematology Department, Hospital de la Santa Creu i Sant Pau and Jose Carreras Leukemia Research Institute, Autonomus University of Barcelona, Barcelona, Spain.; 14 Hospital Universitario la Fe, Hematology Department, Department of Medicine, University of Valencia, Valencia, Spain.; 15 Radboud Institute Molecular Studies, Radboud University Medical Center, Nijmegen, The Netherlands.; 16 Hematology and Oncology, University of Leipzig, Leipzig, Germany.; 17 Department of Hematology and Oncology, Center of Internal Medicine, Otto-von-Guericke University Magdeburg, Magdeburg, Germany.; 18 Medizinische Klinik und Poliklinik I, Universitätsklinikum Carl Gustav Carus der TU Dresden, Dresden, Germany.; 19 Department of Hematology, Hemostasis, Oncology and Stem Cell Transplantation, Hannover Medical School, Hannover, Germany.; 20 Department of Medicine and Comprehensive Cancer Center, University of Chicago, Chicago, IL, USA.; 21 Department of Medical Oncology, Dana-Farber/Partners CancerCare, Boston, MA, USA.

## Abstract

In acute myeloid leukemia (AML) internal tandem duplications of the *FLT3* gene (*FLT3-*ITD) are associated with poor prognosis. Retrospectively, we investigated the prognostic and predictive impact of *FLT3*-ITD insertion site (IS) in 452 patients randomized within the RATIFY trial, which evaluated midostaurin additionally to intensive chemotherapy. Next-generation sequencing identified 908 ITDs, with 643 IS in the juxtamembrane domain (JMD) and 265 IS in the tyrosine kinase domain-1 (TKD1). According to IS, patients were categorized as JMDsole (*n* = 251, 55%), JMD and TKD1 (JMD/TKD1; *n* = 117, 26%), and TKD1sole (*n* = 84, 19%). While clinical variables did not differ among the 3 groups, *NPM1* mutation was correlated with JMDsole (*P* = 0.028). Overall survival (OS) differed significantly, with estimated 4-year OS probabilities of 0.44, 0.50, and 0.30 for JMDsole, JMD/TKD1, and TKD1sole, respectively (*P* = 0.032). Multivariate (cause-specific) Cox models for OS and cumulative incidence of relapse using allogeneic hematopoietic cell transplantation (HCT) in first complete remission as a time-dependent variable identified TKD1sole as unfavorable and HCT as favorable factors. In addition, Midostaurin exerted a significant benefit only for JMDsole. Our results confirm the distinct molecular heterogeneity of *FLT3*-ITD and the negative prognostic impact of TKD1 IS in AML that was not overcome by midostaurin.

## INTRODUCTION

Internal tandem duplications of the *FLT3* gene (*FLT3*-ITD), resulting in duplication of 3 to more than hundreds of nucleotides, are present in ~25% of younger adults with newly diagnosed acute myeloid leukemia (AML) [1–4]. The FLT3 receptor consisting of an extracellular domain of 5 immunoglobulin-like domains, a transmembrane region, a cytoplasmic juxtamembrane domain (JMD), and 2 cytoplasmic tyrosine kinase domains (TKD1 and TKD2), interrupted by a short kinase insert [5], plays an important role in self-renewal and differentiation of hematopoietic stem and progenitor cells [6–8]. The JMD, that can be subdivided into the JM-binding (JM-B) motif, the switch motif (JM-S), the linker/zipper peptide segment (JM-Z), and the hinge region of JMD, exerts a negative regulatory function over TKD1, consisting of beta1-sheet, nucleotide-binding loop, beta2-sheet, and 3óf beta2-sheet, as well as over TKD2 [5]. By disrupting the autoinhibitory function of the JMD or mediating constitutive phosphorylation of the TKDs, ITD mutations within JMD and TKD1, always affecting exons 14 and 15 of *FLT3* and causing in-frame amino acid changes, lead to constitutive activation of the receptor tyrosine kinase and its downstream signaling pathways with consecutive dysregulated cellular proliferation [9–12]. The majority of ITD insertions (70%) occur within the JMD, whereas ITDs outside the JMD integrating in the TKD1 can be detected in ~30% of ITDs most frequently affecting the beta-1 sheet of TKD1 (~25% of all ITDs) [12, 13]. Several studies have shown that ITD mutations are associated with poor prognosis due to a high relapse rate, in particular in cases with a high mutant to wild-type allelic ratio (AR), commonly defined by a threshold of 0.50 using capillary electrophoresis [14–19], and/or insertion site (IS) located in the beta1-sheet of TKD1 [13, 18, 20].

Previous studies have demonstrated that TKD1-ITDs are associated with resistance to chemotherapy and significantly inferior outcome [13, 18, 20]. Here, in vitro studies showed that non-JMD-ITDs confer resistance to a panel of FLT3 inhibitors [21, 22]. In transfection studies performed by Arreba-Tutusaus and colleagues, 32D cells, Ba/F3 cells, and primary mouse bone marrow cells transduced with TKD1-ITD constructs displayed significantly less apoptosis compared to JMD-ITD constructs when exposed to midostaurin, and also quizartinib [22].

The natural course of *FLT3* mutated AML may change with the advent of FLT3 tyrosine kinase inhibitors (TKI) that are now becoming increasingly available. In the CALGB 10603/RATIFY trial, enrolling 717 *FLT3* mutated (ITD and TKD) adult AML patients, the addition of the multikinase inhibitor midostaurin to intensive chemotherapy significantly improved overall survival (OS) and event-free survival (EFS) [23]. The results from the trial led to the approval of midostaurin for first-line treatment of *FLT3* mutated AML in 2017.

The aims of this large, retrospective study were to assess the molecular landscape of *FLT3*-ITD and to evaluate the prognostic impact of ITD IS on OS and cumulative incidence of relapse (CIR) in patients enrolled on the CALGB 10603/RATIFY trial. In addition, the predictive impact of ITD IS for response to treatment with midostaurin was evaluated.

### Patients and methods

Overall, 717 patients with AML and activating *FLT3* mutations (ITD and TKD mutations) were included in the CALGB 10603/RATIFY trial [23]. This exploratory post-hoc analysis focused on the prognostic and predictive impact of ITD IS in the subset of 555 *FLT3*-ITD positive (*FLT3*-ITD+) patients. Identification of *FLT3*-ITDs for study entry was performed by PCR-based amplification of *FLT3* exon 14 and 15 using gene-specific primers followed by capillary electrophoresis (CE); stored biosamples for next-generation sequencing (NGS) analysis were available in 452/555 (81%) patients. Clinical and genetic baseline data of the patients are given in Table 1.

### Molecular analyses

*FLT3*-ITD mutation analysis and assessment of *NPM1* mutational status were performed as previously described [23, 24].

### *FLT3*-ITD Roche 454 next-generation sequencing analysis

To investigate the relationship between ITD IS and patient outcome, Roche 454 NGS analysis was performed that allowed bi-directional sequencing with sequence read length of >500 bp and the identification of ITDs present in less than 5% of cells. A detailed methodological description is provided in the Supplementary Appendix and in Supplementary Figs. 1 and 2. Sequencing raw data were analyzed using the bioinformatics program *getITD* [25].

### Statistical analyses

Complete remission (CR), OS, CIR, and cumulative incidence of death (CID) were defined by standard criteria [19]; responses included all CRs achieved during induction therapy. Overall survival was calculated from the date of randomization to death of any cause. The time to relapse was calculated from the date of first CR to relapse. Patients not having experienced the event of interest at the end of follow-up are censored at the date of last contact. CIR and CID were computed according to the method described by Gray [26]. The median follow-up for survival was calculated using the reverse Kaplan–Meier estimate [27]. Logistic regression and Cox proportional hazards models were used to identify prognostic variables for CR and OS [28]. CIR was analyzed using cause-specific Cox models where death in CR is considered as a competing event. Modeling the time to death in CR was not possible due to a very low number of events (22 events, 19 in the JMDsole group). Additional covariates in multivariate analysis were IS (categorized as JMDsole, JMD/TKD1, and TKD1sole due to the respective functional consequences and in line with previous studies [18, 20]), treatment with midostaurin (vs placebo), and *NPM1* mutation status as dichotomous variables, as well as NGS-based calculated *FLT3*-ITD AR per patients (NGS-cAR) [determined as ∑variant allele frequency (VAF)/(100-∑VAF); log2 transformed], number of ITDs, white blood cell (WBC) count (log2 transformed), and age as continuous variables; allogeneic hematopoietic cell transplantation (HCT) in first CR (CR1) was included as a time-dependent variable. Kruskal-Wallis test was used for comparing quantitative variables between patient subgroups; categorical variables were compared by means of Fisher’s exact test. Associations between continuous variables were analyzed using the Spearman rank correlation coefficient. Survival distributions were estimated using the Kaplan–Meier method, and differences between groups were analyzed using two-sided log-rank tests. An effect was considered significant if its *P* value was less than 5%. The analyses were not adjusted for multiple testing. All statistical analyses were performed with the statistical software R 4.0.2 and/or IBM SPSS Statistics 25.

## RESULTS

### Patient characteristics

Table 1 summarizes the baseline characteristics of the 452 *FLT3*-ITD+ patients. According to treatment arms, patients in the placebo group exhibited more frequently a normal karyotype (80.1% vs 62.2%; *P* < 0.001), *NPM1* mutation (*NPM1*^mut^, 64.3% vs 50.0%; *P* = 0.008), and the favorable genotype *NPM1*^mut^/*FLT3*-ITD^low^ (23.9% vs 14.7%; *P* = 0.031).

### Next-generation sequencing

We identified a total of 908 high confidence *FLT3*-ITDs in the 452 patients by NGS. The median coverage (total number of reads per patient) was 3115 (range: 482–11,616), median ITD counts were 97 (range: 2–5234), median VAF was 3.34% (range: 0.03–89.49%), and the median length was 45 base pairs (bp) (range: 6–246 bp).

### Molecular characterization of *FLT3*-ITDs

**Fig. 1:**
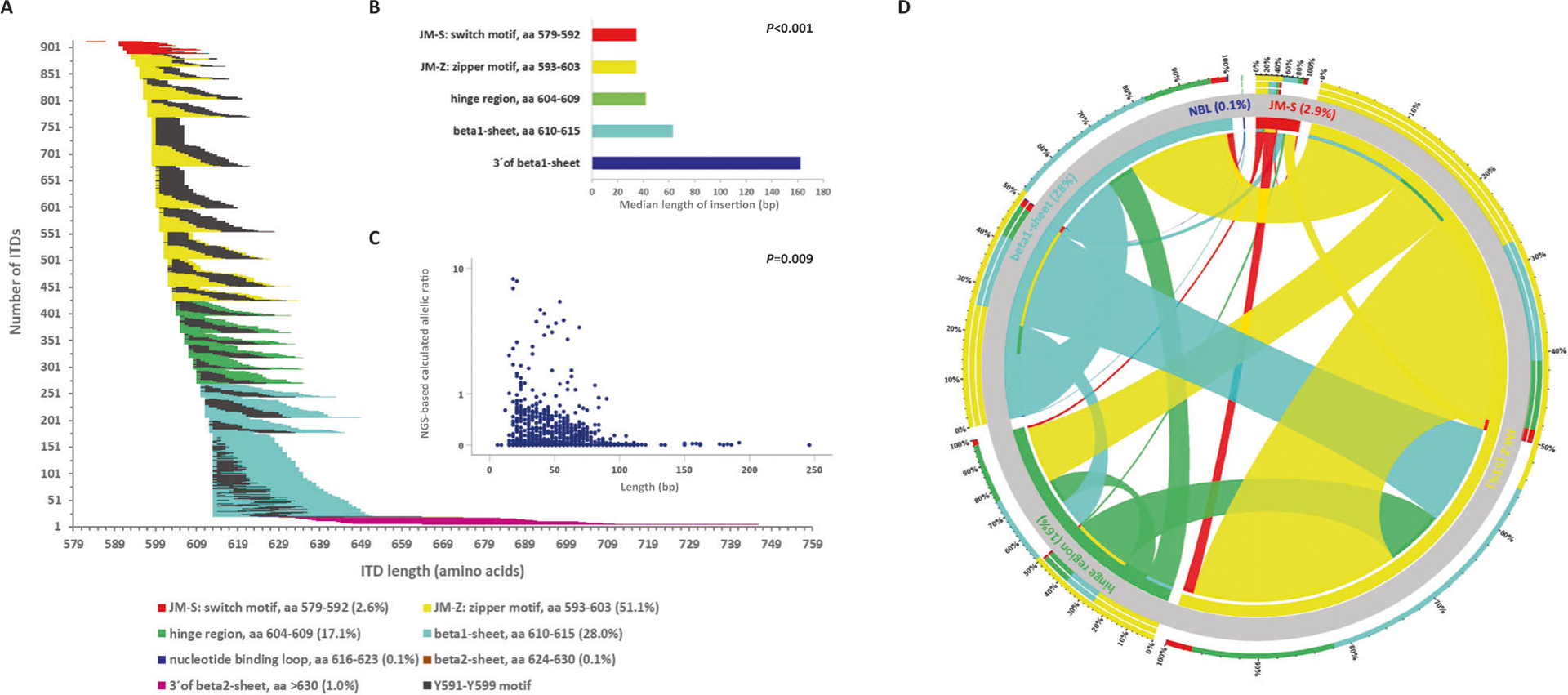
Molecular landscape of *FLT3*-ITD. **A** correlation between ITD IS, length, as well as the relative position of the Y591-Y599 motif (depicted in black) of the 908 ITDs sorted by IS. The duplicated fragments are color-coded according to IS and the black color demonstrates the involvement of the amino acid motif Y591-Y599 which is important for intracellular signaling. The majority of the 908 ITDs included at least a part of the specific amino acid stretch Y591–599. **B** Correlation of ITD IS with length. **C** Correlation of ITD length with NGS-based calculated allelic ratio. **D** Circos Plot showing the interaction of concurrent IS. In 242 pts (53.5%) featuring multiple ITD clones, 698 concurrent IS were delineated; coexistent IS within JMD-Z were the most frequent interaction (41.5%) followed by the interaction between JMD-Z and beta1-sheet (20.2%), within beta1-sheet (12.2%), between JMD-Z and hinge region (11.5%), between beta1-sheet and hinge region (5.5%), and within the hinge region (4.8%).

Two-hundred and ten (46.5%) of 452 *FLT3*-ITD + AML patients had one ITD, and 242 (53.5%) harbored more than one ITD (2 ITDs, *n* = 131 [29%]; 3 ITDs, *n* = 58 [13%]; 4 ITDs, *n* = 24 [5%]; 5 ITDs, *n* = 18 [4%]; 6 ITDs, *n* = 3 [1%]; 7 ITDs, *n* = 7 [2%]; 9 ITDs, *n* = 1 [0.2%]). All ITDs, except of 2 which exclusively affected exon 15, involved exon 14 of *FLT3*, and all ITDs were in-frame with a direct head-to-tail orientation. Eighty-eight ITDs also contained intron 14, and 15 ITDs extended to exon 15. In 619/908 (68.2%) ITDs, insertions resulted in a change of the expected amino acid sequence at the respective site. According to Griffith et al. [5] as well as previous and current releases of the e!Ensembl database (release 103, February 2021: https://www.ensembl.org/Homo_sapiens/Transcript/ProteinSummary?db=core;g=ENSG00000122025;r=13:28003274-28100592;t=ENST00000241453), molecular characterization of the ITDs revealed that in the majority (70.8%, *n* = 643) the IS was localized in the JMD between amino acids (aa) 573 and 609, whereas the remaining ITDs (29.2%) inserted in 3´ direction of the JMD, predominantly in the beta1-sheet of TKD1 between aa 610 and 615 (27.5%, *n* = 250) (Fig. 1A), which is in line with previous studies [12, 13, 18]. *FLT3*-ITD size was significantly correlated with IS, in that the more C-terminal the insertion of the IS, the longer the size of the inserted fragment [Rho (Spearman) = 0.530; *P* < 0.001] (Fig. 1B).

### Analysis of the duplication of specific amino acid motives

Because of the particular role of the tyrosine-rich stretch Y591 to Y599 (YVDFREYEY) for intracellular signaling [29], we determined the frequency of this motif in the ITDs. Duplication of at least one residue in this specific stretch was seen in 840/908 (92.5%) ITDs. The most frequent affected aa residues were R595 in 73.9% (*n* = 671), E596 (73.8%; *n* = 670), and Y597 (73.2%; *n* = 665), followed in descending order by F594 (71.0%; *n* = 645), D593 (68.6%; *n* = 623), E598 (66.1%; *n* = 600), V592 (60.7%; *n* = 551), Y599 (59.8%; *n* = 543), and Y591 (55.0%; *n* = 499). The entire Y591-Y599 motif was duplicated in 265 (29.2%) ITDs. Taking patients as denominator, in 425/452 (94.0%) patients at least one ITD showed the involvement of at least one aa of this specific motif. The entire Y591-Y599 motif was duplicated in 117 (25.9%) patients. In Fig. 1A, length of the ITDs, IS, and relative position of the Y591-Y599 motif within the ITDs are illustrated.

### Mutant to wild-type *FLT3*-ITD allelic ratio determined by NGS (NGS-cAR)

NGS-based quantitative *FLT3*-ITD mutant to wild-type AR (NGS-cAR) was calculated based on VAF as VAF/(100-VAF). The NGS-cAR ranged from 0.0003 to 8.510, with a median of 0.04. The NGS-cAR correlated inversely with ITD length [Rho (Spearman) = −0.087; *P* = 0.009] (Fig. 1C) and aa insertion (5´ to 3´) (Rho = −0.081; *P* = 0.015), in that the higher the NGS-cAR, the shorter the ITD and the more 3´ the insertion site. NGS-cAR per patient [∑VAF/(100-∑VAF)] ranged from 0.0006 to 10.75, with a median of 0.27 and was correlated inversely with the number of ITDs [Rho (Spearman) = −0.142; *P* = 0.002], in that the higher the NGS-cAR, the lower the number of ITD mutated clones.

### Characterization of co-occurring insertion sites

In 242 patients (54%) featuring multiple ITD clones, 698 co-occurring IS were detected. Figure 1D illustrates the relative interaction of concurrent IS. In detail: coexistent IS within JMD-Z were the most frequent interaction (41.5%) followed by the interaction between JMD-Z and beta1-sheet (20.2%), within beta1-sheet (12.2%), between JMD-Z and hinge region (11.5%), between beta1-sheet and hinge region (5.5%), and within the hinge region (4.8%) (Fig. 1D). Furthermore, the vast majority of patients (204/242; 84%) with more than one ITD exhibited one dominant ITD with an NGS-cAR at least twice as high as the sum of the NGS-cARs of the co-insertions (Supplementary Fig. 3).

### Correlation of *FLT3*-ITD IS with clinical baseline characteristics, genetic features, and treatment

We correlated *FLT3*-ITD IS, categorized as patients having IS in (i) JMDsole (*n* = 251, 55.5%), (ii) JMD/TKD1 (*n* = 117, 25.9%), and (iii) TKD1sole (*n* = 84, 18.6%), with age, gender, WBC counts, bone marrow (BM) blast counts, *NPM1*^mut^, *FLT3*-ITD AR (based on Genescan analysis), and the four *NPM1*/*FLT3*-ITD genotypes, as defined within the 2017 European LeukemiaNet (ELN) risk categorization [19], number of ITDs, NGS-cAR, as well as treatment arm (midostaurin vs placebo) and HCT in CR1. While clinical variables did not differ among the 3 groups, JMDsole insertion was positively correlated with *NPM1*^mut^ (*P* = 0.028), inversely with the *NPM1*^wt^/*FLT3*-ITD^low^ genotype (*P* < 0.001), and showed higher AR, determined by both Genescan and NGS-based (*P* < 0.001, each). Patients with JMD/TKD1 insertions exhibited more frequently *FLT3*-ITD^low^ (AR ≤ 0.5) (*P* < 0.001) and had more ITD subclones (*P* < 0.001). Of note, treatment with midostaurin was more frequently performed in patients with TKD1sole insertion (*P* = 0.012) (Table 2).

### Clinical outcome

#### Response to induction therapy.

**Fig. 2:**
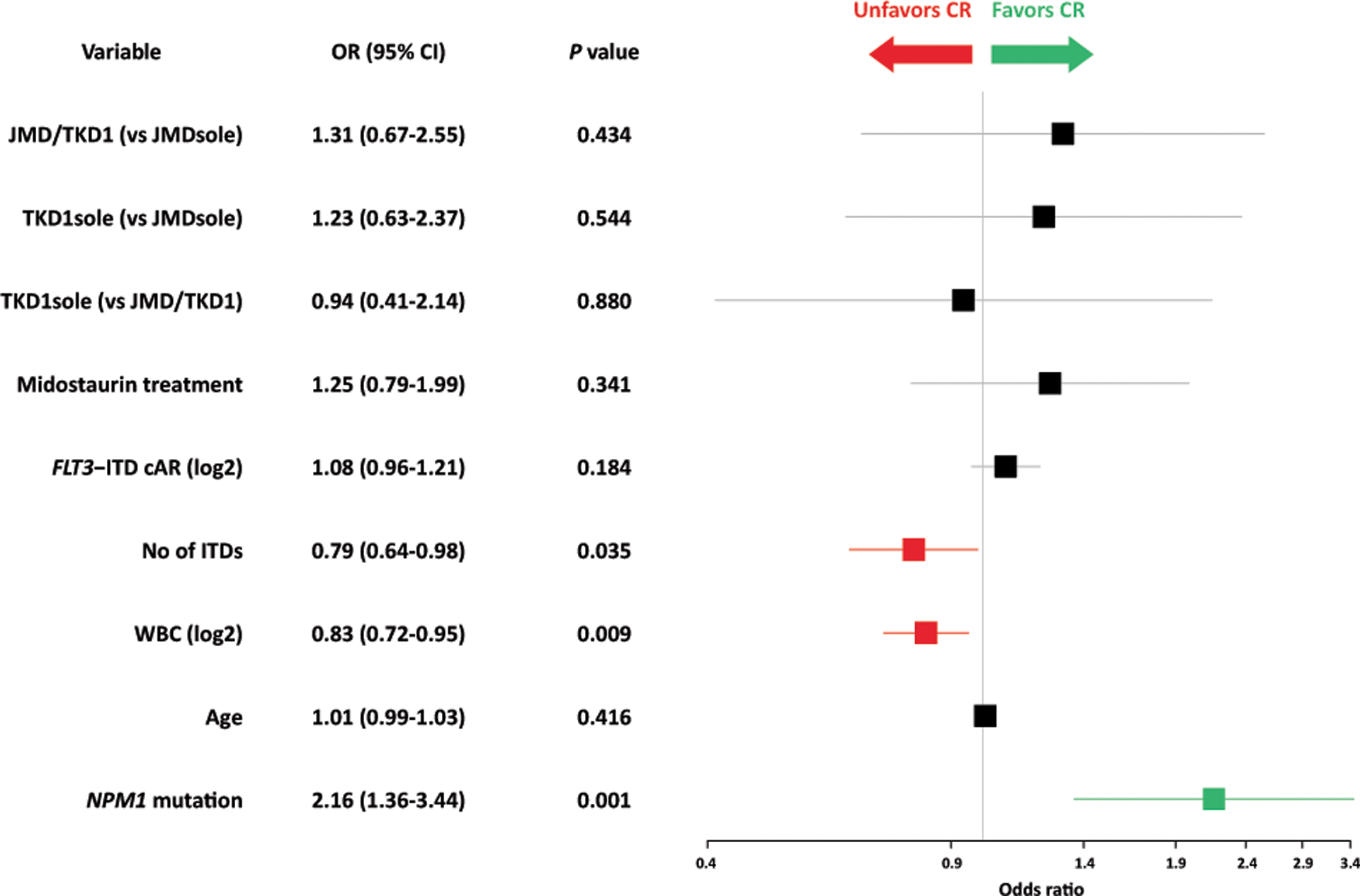
Forest plot of Odds ratios for response to induction therapy derived by multivariate analysis by various clinical and genetic variables. OR Odds ratio, CI confidence interval, cAR NGS-based calculated allelic ratio.

Complete remission, including all CRs during induction cycles 1 and 2, was achieved in 274/452 (60.6%) patients. To address the impact of IS on CR, correlations of the 3 categorized IS groups [(i) JMDsole, (ii) JMD/TKD1, and (iii) TKD1sole] were performed using a logistic regression model. This model revealed *NPM1*^mut^ as a favorable marker for achievement of CR (OR, 2.16; 95% confidence interval [CI], 1.36–3.44; *P* = 0.001), while WBC (OR for doubling of WBC, 0.83; 95% CI, 0.72–0.95; *P* = 0.009) and number of ITDs (OR, 0.79; 95% CI, 0.64–0.98; *P* = 0.035) predicted for a lower CR rate. The 3 IS groups as well as other variables had no impact on CR achievement (Fig. 2).

#### Survival analysis.

The estimated median follow-up of the 452 *FLT3*-ITD + AML patients was 60.6 months (95% CI, 57.4–62.9); 248 of the 452 (54.9%) patients died. Median OS time and 4-year OS rate were 24.4 months (95% CI, 18.6–35.9 months) and 0.43 (95% CI, 0.38–0.47), respectively. HCT in CR1 was performed in 99/452 (21.9%) patients, and overall during the disease course in 257/452 (56.9%) patients.

**Fig. 3:**
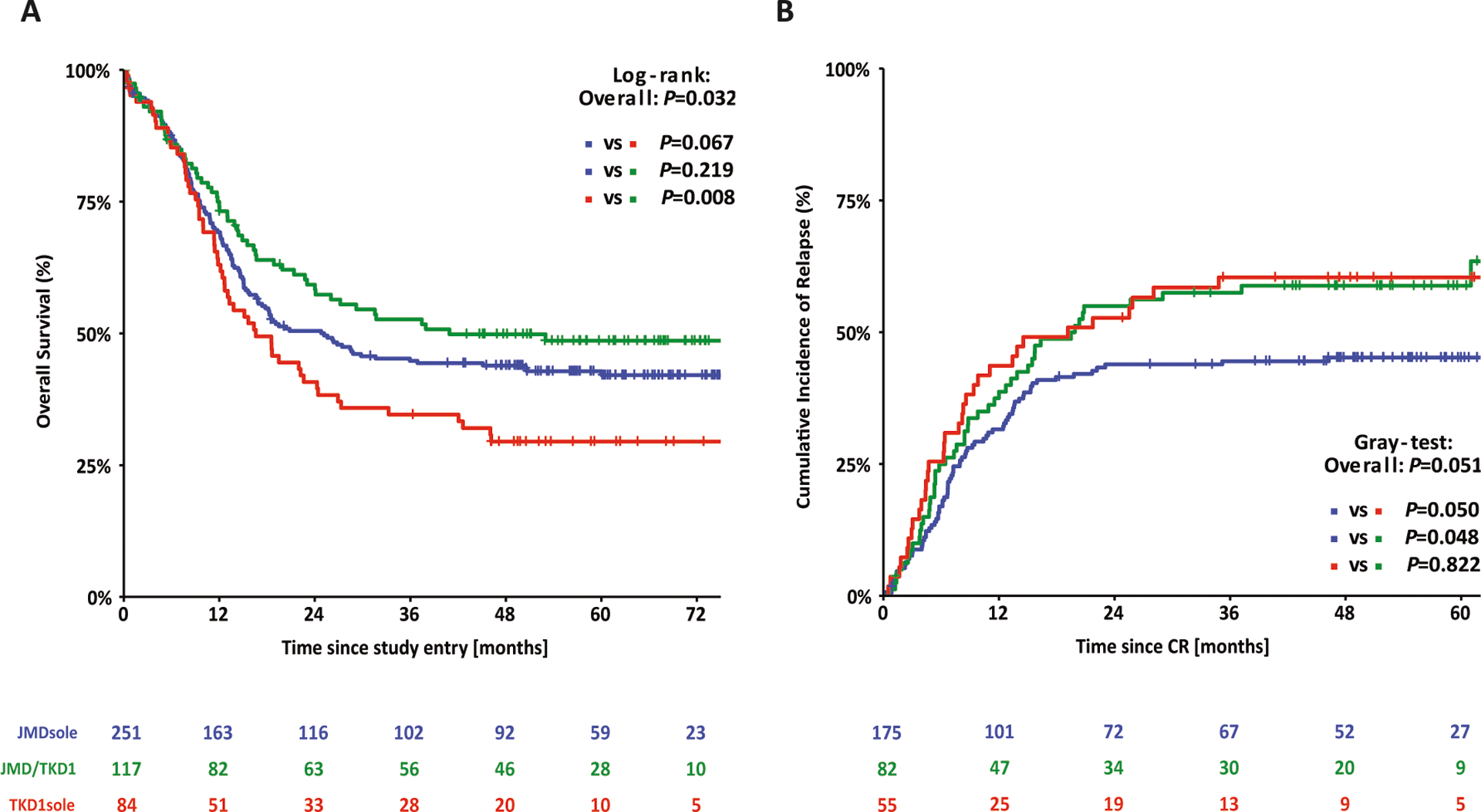
Survival analysis for *FLT3*-ITD + AML according to ITD IS. Kaplan–Meier curves for overall survival (**A**) and cumulative incidence of relapse (**B**) for the 452 *FLT3*-ITD + AML patients according to ITD IS.

Survival analyses according to ITD IS showed that patients with insertions in TKD1sole had a significantly inferior OS and a higher CIR compared to patients with insertion sites in JMDsole and/or JMD/TKD1 (4-year OS rates TKD1sole vs JMDsole vs JMD/TKD1: 29% vs 44% vs 50%; *P* = 0.032; 4-year CIR: 60% vs 45% vs 59%; *P* = 0.051) (Fig. 3). Supplementary Figure 4 shows CID according to ITD IS. In the JMD/TKD1 group, there was a significantly lower CID rate (4-year CID rates JMD/TKD1 vs TKD1sole vs JMDsole: 4% vs 13% vs 15%; *P* = 0.027), providing an explanation for the discrepancy between improved OS without decreasing the relapse rate for this subgroup.

In Cox regression analyses on OS, *FLT3*-ITD IS in TKD1sole (compared to JMDsole: HR, 1.61; 95% CI, 1.10–2.34; *P* = 0.014, and compared to JMD/TKD1: HR, 2.17; 95% CI, 1.29–3.67; *P* = 0.004), higher WBC (log2) (HR, 1.12; 95% CI, 1.03–1.23; *P* = 0.010), and increasing age (HR, 1.17; 95% CI, 1.02–1.43; *P* = 0.029) revealed as unfavorable factors for OS, whereas *NPM1*^mut^ (HR, 0.58; 95% CI, 0.43–0.78; *P* < 0.001) and HCT in CR1 (HR, 0.46; 95% CI, 0.30–0.70; *P* < 0.001) were both favorable. Multiclonality (higher number of ITDs) (HR, 1.13; 95% CI, 0.99–1.28; *P* = 0.076) and higher NGS-cAR (HR, 1.07; 95% CI, 0.99–1.15; *P* = 0.093) were in trend associated with inferior OS. Treatment with midostaurin had no additional impact on OS. For CIR, *FLT3*-ITD IS in TKD1sole (compared to JMDsole: HR, 2.20; 95% CI, 1.34–3.59; *P* = 0.002), higher WBC (log2) (HR, 1.18; 95% CI, 1.06–1.31; *P* = 0.003), and higher NGS-cAR (HR, 1.16; 95% CI, 1.04–1.29; *P* = 0.007) were associated with higher risk of relapse, whereas *NPM1*^mut^ (HR, 0.67; 95% CI, 0.45–0.98; *P* = 0.037) and HCT in CR1 (HR, 0.42; 95% CI, 0.26–0.69; *P* < 0.001) reduced risk of relapse significantly. Treatment with midostaurin again had no impact. The results of the Cox regression analyses are summarized in Table 3.

**Fig. 4:**
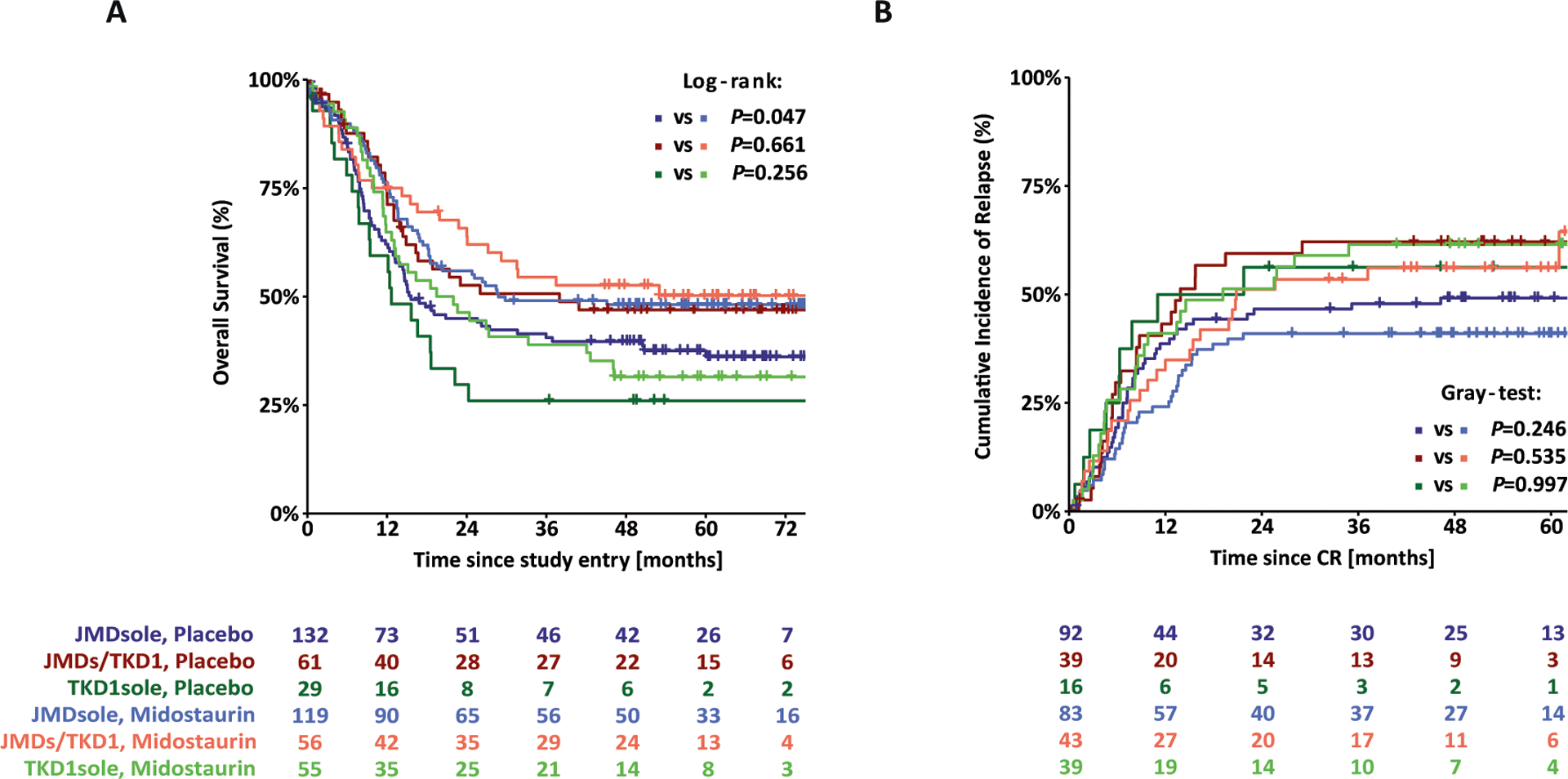
Survival analysis according to ITD IS and to treatment arm. Kaplan–Meier curves for overall survival (**A**) and cumulative incidence of relapse (**B**) for the 452 *FLT3*-ITD + AML patients according to ITD IS and to treatment arms (midostaurin vs placebo).

Survival analyses for OS and CIR according to ITD IS and to treatment arms are shown in Fig. 4. The 4-year rates for OS of patients on midostaurin or placebo were 48% vs 40% (*P* = 0.047) for JMDsole, 53% vs 47% (*P* = 0.661) for JMD/TKD1, and 32% vs 26% (*P* = 0.256) for TKD1sole subgroups, respectively. For CIR, the 4-year rates for patients on midostaurin or placebo were 40% vs 50% (*P* = 0.246) for JMDsole, 56% vs 63% (*P* = 0.535) for JMD/TKD1, and 56% vs 63% (*P* = 0.997) for TKD1sole subgroups, respectively.

We next performed Cox regression analyses on OS and CIR according to the 3 IS subgroups (Table 3). Within the JMDsole group, NGS-cAR (HR for OS, 1.18; 95% CI, 1.05–1.33; *P* = 0.006 and HR for CIR, 1.45; 95% CI, 1.21–1.73; *P* < 0.001) and multiclonality (HR, 1.29; 95% CI, 1.04–1.60; *P* = 0.022 and HR, 1.43; 95% CI, 1.02–2.02; *P* = 0.040) had a negative impact on both OS and CIR, older age (HR, 1.24; 95% CI, 1.03–1.51; *P* = 0.024) on OS only, whereas treatment with midostaurin (HR, 0.56; 95% CI, 0.37–0.84; *P* = 0.005 and HR, 0.57; 95% CI, 0.34–0.96; *P* = 0.036) and *NPM1*^mut^ (HR, 0.47; 95% CI, 0.31–0.72; *P* < 0.001 and HR, 0.46; 95% CI, 0.27–0.78; *P* = 0.004) were of beneficial impact on both endpoints. A significant beneficial effect for HCT in CR1 was observed only for CIR (HR, 0.40; 95% CI, 0.20–0.80; *P* = 0.010). Within the TKD1sole group, HCT in CR1 (HR, 0.28; 95% CI, 0.12–0.68; *P* = 0.005 and HR, 0.19; 95% CI, 0.06–0.57; *P* = 0.003) was the only significant factor for improved OS and CIR, whereas higher WBC (log2) (HR, 1.38; 95% CI, 1.09–1.75; *P* = 0.008 and HR, 1.33; 95% CI, 1.04–1.70; *P* = 0.021) were of adverse impact for OS and CIR. The only significant factor within the JMD/TKD1 group was HCT in CR1 (HR, 0.17; 95% CI, 0.04–0.74; *P* = 0.018) associated with improved OS.

To further explore the prognostic impact of concurrent *NPM1*^mut^, we evaluated OS and CIR according to ITD IS with or without concurrent *NPM1*^mut^ and to treatment arms. The best outcome was observed for the genotype JMDsole/*NPM1*^mut^ additionally treated with midostaurin. The 4-year rates for OS and CIR for this genotype on midostaurin or placebo were 65% vs 44% (*P* = 0.016) and 30% vs 53% (*P* = 0.018), respectively. The only other genotype benefitting in trend from additional midostaurin was JMDsole/*NPM1*^wt^ with 4-year rates for OS and CIR (midostaurin vs placebo) of 40% vs 28% (*P* = 0.075) and 53% vs 63% (*P* = 0.319), respectively. There was no benefit for additional midostaurin treatment within the other genotypes (Supplementary Figure 5). Of note, patient numbers in other genetic subgroups were relatively small and the study was not powered to perform subgroup analysis. Therefore, additional genotype-specific subgroup analyses (e.g., *DNMT3A*, *RUNX1*, etc.) were refrained since this will result in splitting up in small subgroups not allowing sound and meaningful statistical analyses.

## DISCUSSION

The results from this retrospective explorative analysis of the RATIFY trial confirm the distinct molecular heterogeneity of *FLT3*-ITD and the negative prognostic impact of TKD1 insertions. Furthermore, the data show that this negative impact might not be overcome by treatment with the multikinase inhibitor midostaurin confirming data from earlier preclinical studies. Additional midostaurin exerted a significant beneficial effect in patients with IS in JMDsole, in particular with concurrent *NPM1*^mut^, whereas no significant benefit was seen in the other patient groups. In multivariable analysis TKD1sole insertion revealed as an unfavorable prognostic factor for OS and CIR, whereas *NPM1*^mut^ and HCT in CR1 were associated with improved outcome.

In this large cohort of 452 younger adult AML patients, we could confirm that ~30% of *FLT3*-ITDs are localized outside the JMD with a major cluster in the beta1-sheet of the TKD1 (28%), and at very low frequencies (≤1%) in the nucleotide-binding loop, the beta2-sheet of the TKD1, and 3´ of the beta2-sheet [12, 13]. As previously described, ITD size also strongly correlated with IS, in that the more C-terminal the insertion, the longer the inserted fragment [13]. Of note, NGS-cAR correlated inversely with (i) ITD length, (ii) the genomic insertion (aa, 5´to 3´), and (iii) the number of ITDs per patient. While the inverse correlation between NGS-cAR and the collinear ITD length and genomic insertion (the higher the NGS-cAR, the shorter the ITD and the more 5´ the IS) might be caused by the amplification disadvantage of larger DNA fragments, the inverse correlation between NGS-cAR and number of ITDs per patient remains elusive. In addition, in this cohort the majority of *FLT3*-ITD + AML patients (54%) harbored more than one ITD of different leukemic subclones which is related to the higher sensitivity of NGS compared to conventional DNA fragment analyses [25]; 84% of these patients exhibited one dominant *FLT3*-ITD clone with an NGS-cAR at least twice as high as the sum of the NGS-cARs of the co-occurring ITDs. In line with a previous report [13], the vast majority of patients (92%) with >1 ITD exhibited at least one insertion within the JMD.

*FLT3*-ITD IS were not associated with defined clinical characteristics, but with distinct genetic features: concurrent *NPM1*^mut^ correlated positively with IS in JMDsole, and *FLT3*-ITD low AR (determined by fragment analysis) as well as number of ITDs with the JMD/TKD1 subgroup, respectively. Moreover, we observed a significant difference with respect to the 4 *NPM1*/*FLT3*-ITD genotypes: The genotype *NPM1*^wt^/*FLT3*-ITD^low^ was significantly underrepresented in the JMDsole subgroup while being highest in the JMD/TKD1 group (Table 2).

With respect to response to induction therapy the only favorable variable was *NPM1*^mut^ known to predict for achievement of CR, also in AML with concurrent *FLT3*-ITD [13, 18]. Unfavorable factors were higher WBC counts and a higher number of ITD clones per patient (Fig. 2). This finding is at variance to previous studies that showed no impact for number of ITDs on response in multivariate analyses [13, 18]. One possible explanation might be the higher sensitivity of NGS-based ITD subclone detection as supported by the high number of patients with >1 ITD in this study compared to conventional fragment analysis used in the previous studies (54% vs 14%; *P* < 0.001).

The first important clinical finding of this study was the confirmation of the negative prognostic impact of TKD1 insertions [13, 18, 20]. Multivariate analysis for OS using Cox regression in the entire patient cohort identified TKD1sole IS, higher WBC counts, and older age as significant unfavorable factors. Significant favorable variables were *NPM1*^mut^ and HCT in CR1 (Table 3). In the multivariate model for hazard of relapse, TKD1sole IS, higher NGS-cAR, and higher WBC counts predicted for higher risk of relapse, while HCT in CR1 reduced relapse risk (Table 3). Next, we performed multivariate analysis for OS and CIR within the 3 IS groups. The only variable that showed a consistent favorable effect across all subgroups was HCT in CR1 (with the exception of JMD/TKD1 for CIR). Of note, in the JMDsole group treatment with midostaurin – besides *NPM1*^mut^ – had a strong beneficial effect on both OS and CIR (Table 3).

**Fig. 5:**
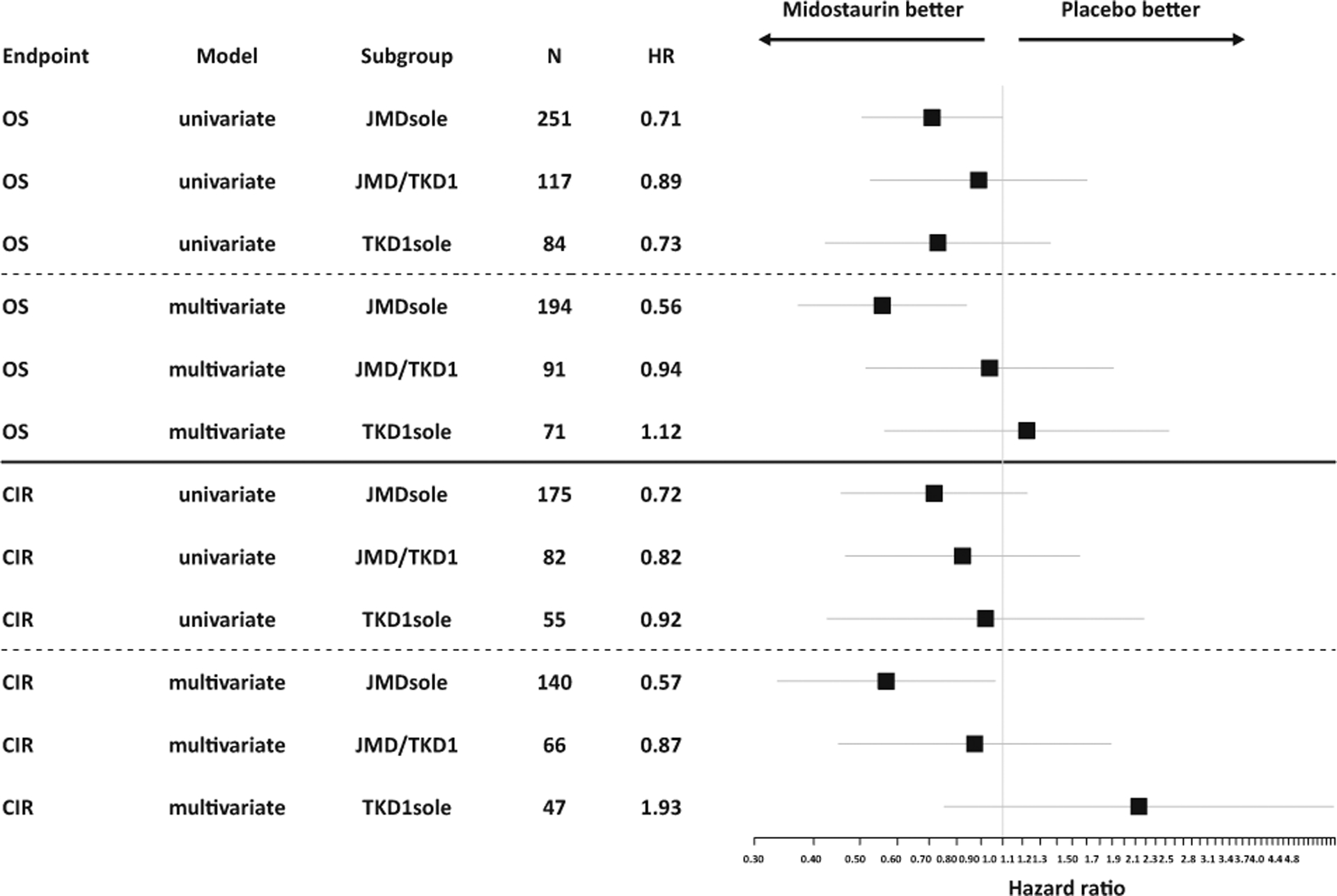
Forest plot of HRs for midostaurin effect on outcome derived from univariate and multivariate Cox models according to IS. JMDsole, JMD/TKD1, TKD1sole.

The second importing finding is that the beneficial effect of midostaurin was restricted to the JMDsole subgroup (Table 3 and Fig. 5) being in line with preclinical data showing sensitivity to midostaurin for JMD-ITD constructs in vitro and in vivo [22]. This benefit might be strengthened by the significant underrepresentation of the genotype *NPM1*^wt^/*FLT3*-ITD^low^, that appears to have no benefit from midostaurin [30], in the JMDsole subgroup (Table 2) as well as by our data illustrating the impact of concurrent *NPM1*^mut^ on OS and CIR in the 3 IS groups and by treatment arm (midostaurin vs placebo) (Supplementary Fig. 5). However, these results need to be interpreted with caution because the RATIFY trial was not powered to show statistically significant differences in these genetic subgroups. Nevertheless, beside the 2017 ELN risk groups our data provide evidence for additional risk stratification based on *FLT3*-ITD IS.

The third interesting finding is that the negative prognostic impact of TKD1 IS was not overcome by additional treatment with midostaurin (Table 3, Figs. 4, 5, and Supplementary Fig. 5). The clinical observation that patients with insertion in TKD1 might not benefit from additional midostaurin does not only confirm the known resistance to chemotherapy of patients with TKD1 insertions [13, 18, 20], but also to TKI treatment, which is in line with preclinical data [21, 22]. Primary resistance of TKD1 insertions to midostaurin was first described in 2009 [21]. In a reconstitution model it could be demonstrated that TKD1-ITD627E was sufficient to confer resistance to a panel of FLT3 TKIs in vitro [21]. Subsequent transfection studies using 32D cells, Ba/F3 cells, and primary mouse bone marrow cells transduced with TKD1-ITD constructs revealed significantly reduced apoptosis for TKD1-ITD constructs compared to JMD-ITD constructs, when exposed to the multikinase inhibitor midostaurin, and also to the highly selective FLT3-inhibitor quizartinib [22]. Mechanisms mediating primary resistance of TKD1-ITDs to TKIs being discussed are upregulation of the anti-apoptotic myeloid cell leukemia 1 protein (MCL-1) and effective DNA-damage repair in TKD insertions possibly protecting from targeted and cytotoxic treatment [21, 22]. Furthermore, the significantly higher CIR rate for patients with TKD1 insertions compared to patients with JMDsole insertions might reflect different patterns of clonal evolution and acquired co-mutations mediating secondary resistance to TKIs between these groups. Complex changes in clonal architecture underlying response and resistance to TKI treatment have recently been described for quizartinib and also gilterinitib [31, 32].

In conclusion, the results from this retrospective analysis of the RATIFY trial further classify the molecular landscape of *FLT3*-ITD. Using a more sensitive NGS approach we identified more than one ITD clone in the majority of *FLT3*-ITD + AML patients. Moreover, our data confirm the negative prognostic impact of TKD1 IS that was not significantly affected by treatment with the multikinase inhibitor midostaurin. A beneficial effect of midostaurin was only found for patients with JMDsole IS. In this subset, *NPM1*^mut^ also exerted a strong beneficial effect.

## Supplementary Material

1755121_Sup_material

## Figures and Tables

**Table 1.** Baseline characteristics of the 452 *FLT3*-ITD positive patients.

	All patients (*n* = 452)	Midostaurin group (*n* = 230)	Placebo group (*n* = 222)	*P* value
Age, years				0.090
Median	47	47	48	
Range	18–60	19–59	18–60	
Male sex, *n*/total *n* (%)	206 (45.6)	114/230 (49.6)	92/222 (41.4)	0.090
WBC count, ×10^9^/l				0.497
Median	42.4	42.6	42.1	
Range	0.8–329.8	0.8–304	0.8–329.8	
Platelet count, ×10^9^/l				0.910
Median	50	50.5	49.5	
Range	2.0–461	2.0–461	8.0–342	
Bone marrow blasts, %				0.47
Median	79	77	80	
Range	3–100	3–100	6–100	
Karyotype, *n*/total *n* (%)				<0.001
Normal	248/348 (71.3)	107/172 (62.2)	141/176 (80.1)	
Abnormal	100/348 (28.7)	65/172 (37.8)	35/176 (19.9)	
2017 ELN Risk Groups, *n*/total *n* (%)				0.138
Favorable	77/268 (28.7)	34/135 (25.2)	43/133 (32.3)	0.225
Intermediate	95/268 (35.4)	45/135 (33.3)	50/133 (37.6)	0.524
Adverse	96/268 (35.8)	56/135 (41.5)	40/133 (30.1)	0.057
*NPM1* mutation, *n*/total *n* (%)	203/358 (56.7)	95/190 (50.0)	108/168 (64.3)	0.008
Subtype of FLT3-ITD, *n*/total *n* (%)				0.700
ITD with low AR (≤0.5)^a^	161/452 (35.7)	80/230 (34.8)	81/222 (36.6)	
ITD with high AR (>0.5)^a^	290/452 (64.3)	149/230 (65.2)	141/222 (63.4)	
*NPM1/FLT3*-ITD genotypes *n*/total *n* (%)				0.030
*NPM1*^mut^/*FLT3*-ITD^low^	68/357 (19.0)	28/190 (14.7)	40/167 (23.9)	0.031
*NPM1*^mut^/*FLT3*-ITD^high^	135/357 (37.8)	67/190 (35.3)	68/167 (40.7)	0.325
*NPM1*^wt^/*FLT3*-ITD^low^	57/357 (16.0)	37/190 (19.5)	20/167 (12.0)	0.060
*NPM1*^wt^/*FLT3*-ITD^high^	97/357 (27.2)	58/190 (30.5)	39/167 (23.4)	0.153

*WBC* white blood cell, *ELN* European LeukemiaNet, *AR* allelic ratio.

a Assessed using Genescan analysis.

**Table 2.** Patient and disease characteristics as well as molecular characterization, 2017 ELN risk stratification, and treatment by ITD insertion site.

	JMDsole (*n* = 251)	JMD/TKD1 (*n* = 117)	TKD1sole (*n* = 84)	*P* value
Age, median (years)	48.3	48.1	46.3	0.171
Gender, male (%)	41.0	48.7	54.8	0.067
WBC, median (range), ×10^9^/l	40.0 (0.8–329.8)	39.9 (0.9–205.4)	53.9 (1.2–144.8)	0.254
BM blasts, median (%)	79.5	75.5	80	0.489
*NPM1*^mut^, *n*/total *n* (%)	123/195 (63.1)	46/92 (50)	34/71 (47.9)	0.028
*FLT3*-ITD AR risk stratification				
*FLT3*-ITD^high^ (AR > 0.5)^a^, *n*/total *n* (%)	178/251 (70.9)	56/117 (47.9)	56/84 (66.7)	<0.001
*NPM1*/*FLT3*-ITD genotypes				0.003
*NPM1*^mut^/*FLT3*-ITD^low^, *n*/total *n* (%)	39/195 (20.0)	19/91 (20.9)	10/71 (14.1)	0.494
*NPM1*^mut^/*FLT3*-ITD^high^, *n*/total *n* (%)	84/195 (43.1)	27/91 (29.7)	24/71 (33.8)	0.069
*NPM1*^wt^/*FLT3*-ITD^low^, *n*/total *n* (%)	18/195 (9.2)	26/91 (28.6)	13/71 (18.3)	<0.001
*NPM1*^wt^/*FLT3*-ITD^high^, *n*/total *n* (%)	54/195 (27.7)	19/91 (20.9)	24/71 (33.8)	0.190
2017 ELN risk groups				0.473
Favorable, *n*/total *n* (%)	41/149 (27.5)	24/67 (35.8)	12/52 (23.1)	0.277
Intermediate, *n*/total *n* (%)	57/149 (38.3)	19/67 (28.4)	19/52 (36.5)	0.366
Adverse, *n*/total *n* (%)	51/149 (34.2)	24/67 (35.8)	21/52 (40.4)	0.709
No of ITDs				
Median	1	3	1	<0.001
Range	1–5	2–9	1–3	
NGS-based *FLT3*-ITD cAR				
Median	0.41	0.19	0.14	<0.001
Range	0.001–10.76	0.003–4.62	0.001–2.80	
Treatment				
Placebo, *n* (%)	132 (52.6)	61 (52.1)	29 (34.5)	0.012
Midostaurin, *n* (%)	119 (47.4)	56 (47.9)	55 (65.5)	
Allogeneic HCT in CR1, *n* (%)	56 (22.3)	22 (18.8)	21 (25.0)	0.562

*WBC* white blood cell, *BM* bone marrow, *AR* allelic ratio, *ELN* European LeukemiaNet.

a Assessed using Genescan analysis.

**Table 3.** Cox regression models on overall survival and cumulative incidence of relapse for all patients and according to IS groups.

	All patients		JMDsole		JMD/TKD1		TKD1sole	
	HR (95% CI)	*P*	HR (95% CI)	*P*	HR (95% CI)	*P*	HR (95% CI)	*P*
Overall survival	*n* = 356		*n* = 194		*n* = 91		*n* = 71	
TKD1sole (vs JMDsole)	1.61 (1.10–2.34)	0.014						
TKD1sole (vs JMD/TKD1)	2.17 (1.29–3.67)	0.004						
JMDsole (vs JMD/TKD1)	1.35 (0.86–2.12)	0.189						
Midostaurin treatment	0.79 (0.59–1.06)	0.120	0.56 (0.37–0.84)	0.005	0.94 (0.52–1.71)	0.835	1.12 (0.56–2.24)	0.741
*FLT3-*ITD cAR (log2)	1.07 (0.99–1.15)	0.093	1.18 (1.05–1.33)	0.006	1.12 (0.93–1.34)	0.239	0.97 (0.87–1.09)	0.641
No of ITDs	1.13 (0.99–1.28)	0.076	1.29 (1.04–1.60)	0.022	1.10 (0.91–1.32)	0.317	1.01 (0.55–1.85)	0.983
WBC (log2)	1.12 (1.03–1.23)	0.010	1.02 (0.91–1.14)	0.767	1.09 (0.89–1.32)	0.408	1.38 (1.09–1.75)	0.008
Age (10-year difference)	1.17 (1.02–1.34)	0.029	1.24 (1.03–1.51)	0.024	0.92 (0.68–1.23)	0.557	1.28 (0.96–1.70)	0.096
*NPM1* mutation	0.58 (0.43–0.78)	<0.001	0.47 (0.31–0.72)	<0.001	0.69 (0.36–1.30)	0.248	0.57 (0.28–1.15)	0.115
HCT in CR1	0.46 (0.30–0.70)	<0.001	0.65 (0.38–1.10)	0.111	0.17 (0.04–0.74)	0.018	0.28 (0.12–0.68)	0.005
Cumulative Incidence of Relapse	*n* = 253		*n* = 140		*n* = 66		*n* = 47	
TKDIsole (vs JMDsole)	2.20 (1.34–3.59)	0.002						
TKDIsole (vs JMD/TKD1)	1.66 (0.90–3.08)	0.107						
JMDsole (vs JMD/TKD1)	0.76 (0.41–1.30)	0.312						
Midostaurin treatment	0.80 (0.56–1.15)	0.222	0.57 (0.34–0.96)	0.036	0.87 (0.45–1.69)	0.686	1.93 (0.75–4.97)	0.171
*FLT3-*ITD cAR (log2)	1.16 (1.04–1.29)	0.007	1.45 (1.21–1.73)	<0.001	1.05 (0.83–1.32)	0.649	1.02 (0.88–1.21)	0.776
No of ITDs	1.05 (0.89–1.24)	0.538	1.43 (1.02–2.02)	0.040	1.02 (0.83–1.26)	0.846	0.69 (0.30–1.58)	0.379
WBC (log2)	1.18 (1.06–1.31)	0.003	1.16 (0.99–1.35)	0.062	1.18 (0.95–1.47)	0.139	1.33 (1.04–1.70)	0.021
Age (10-year difference)	1.00 (0.84–1.18)	0.982	1.04 (0.81–1.33)	0.766	0.98 (0.70–1.37)	0.908	1.02 (0.70–1.50)	0.904
