# Supplementary material for: Molecular landscape and prognostic impact of FLT3-ITD insertion site in acute myeloid leukemia: RATIFY study results
Source: Leukemia. Author manuscript; Available in PMC 2022 Jan 6. (PMC8727286; doi:10.1038/s41375-021-01323-0)
Supplement: 1755121_Sup_material [file NIHMS1755121-supplement-1755121_Sup_material.docx]

**Supplementary Appendix**

**Description of the Roche 454 *FLT3*-ITD sequencing method**

The 454 sequencing method included amplification of the regions of interest in genomic DNA using PCR, followed by purification and quantification, amplicon pooling, emulsion PCR (ePCR), 454 sequencing, and data analysis. Fifty (50) ng of genomic DNA (extracted from mononuclear cells from RATIFY patients) was used for each PCR reaction. The generated amplicon libraries were quality checked and pooled for 454 ePCR for sequencing. Sample data from valid runs that passed data QC and run validity checks were analyzed for *FLT3*-ITD variants as compared to the wild type sequence.

There were two independent reactions in the assay for ITD that each targeted one of the two overlapping target regions for the ITD of 209 bp (noted as 1st half ITD or ITD1) and 219 bp (noted as 2nd half of ITD or ITD2) of the *FLT3* gene, respectively. The final ITD result was the combined result from 1st half ITD and 2nd half ITD reactions. Data were evaluated using the standard 454 sequencing quality criteria for usable reads.

**Supplementary Figure 1: 454 Sequencing Assay Workflow**

**DNA from Patients**

Sample QC

PCR/barcoding for ITD1

PCR/barcoding for ITD2

PCR amplicon library purification, QC and pooling

454 Sequencing

Sequencing data analysis (*Get*ITD)

Number of ITD

ITD insertion length

Insertion site (IS)

Combining results from ITD1 and ITD2

***FLT3*-ITD sequencing targets**

The *FLT3*-ITD sequencing method was designed to amplify a region of the *FLT3* juxtamembrane domain to identify the presence of internal tandem duplications that occur anywhere in the target region depicted below (exons 14 and 15 are shown in red; intron sequences are shown in black). The ITD target region was split into two overlapping amplicons ITD1 and ITD2 (209 bp and 219 bp, respectively) to optimize the identification of large tandem duplications. (PCR primer sites for ITD1 are highlighted in green, for ITD2 in magenta)

AACTGACTCATCATTTCATCTCTGAAgcaatttaggtatgaaagccagctacagatggtacaggtgaccggctcctcagataatgagtacttctacgttgatttcagagaatatgaatatgatctcaaatgggagtttccaagagaaaatttagagtttggtaagaatggaatgtgccaaatgtttctgcagcatttcttttccattggaaaatctttaaaatgcacgtactcaccatttgtctttgcagggaaggtactaggatcaggtgcttttggaaaagtgatgaacgcaacagcttatggaattagcaaaacaggagtctcaatccaggttgccgtcaaaatgctgaaagGTACAGTATAGTGGAAGGACAGCA

**Sample testing and primers used for multiplexing**

Sample testing with 454 sequencing was conducted by Clarient Diagnostics, Inc. at SeqWright site (Houston, TX, USA). The assay used Roche 454 GS FLX Titanium Next Generation Sequencing platform. Qualified DNA samples were PCR amplified using target specific primers. Multiplexing was accomplished by using unique primer sets for each sample and type of reaction, which include sequences serving as sequencing primer, a 10 nucleotide molecular barcode and amplicon primer. An example of a primer pair used for a sample for the ITD1 reaction is shown in Figure S2. For each sample testing run, DNA from cell lines or plasmid containing ITD was used as positive control, and DNA from cell line with no ITD as negative control. **Supplementary Figure 2: Example primer sequences**

**Forward primer for ITD1** CGTATCGCCTCCCTCGCGCCATCAGACGCTCGACAAACTGACTCATCATTTCATCTCTG

*Sequencing primer* *Barcode* *ITD1 target primer*

**Reverse primer for ITD1** ATGCGCCTTGCCAGCCCGCTCAGACGCTCGACACCAATGGAAAAGAAATGCTGCAG

**Supplementary Figure 3.** Distribution of *FLT3*-ITD clone-specific allelic ratio in patients with >1 ITD.


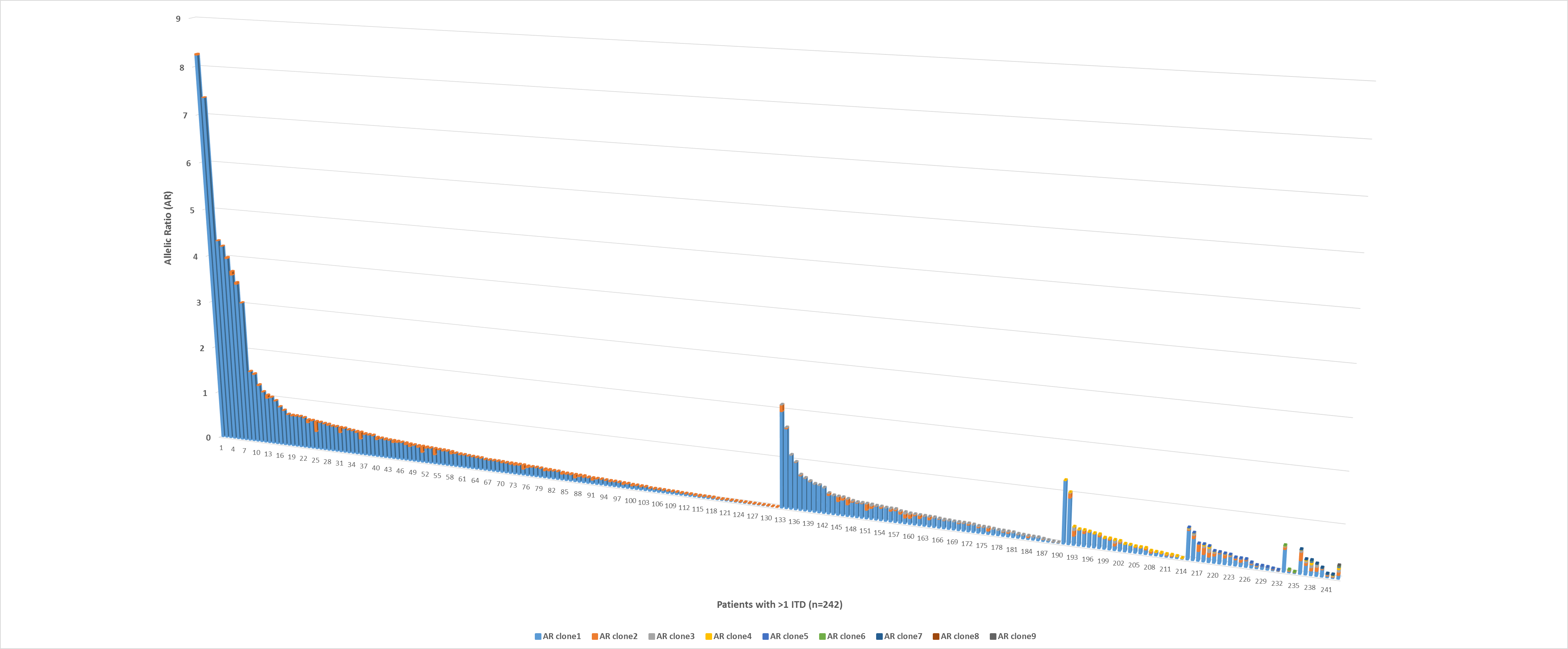


**Supplementary Figure 4.** Cumulative incidence of death of patients achieved complete remission after induction therapy.


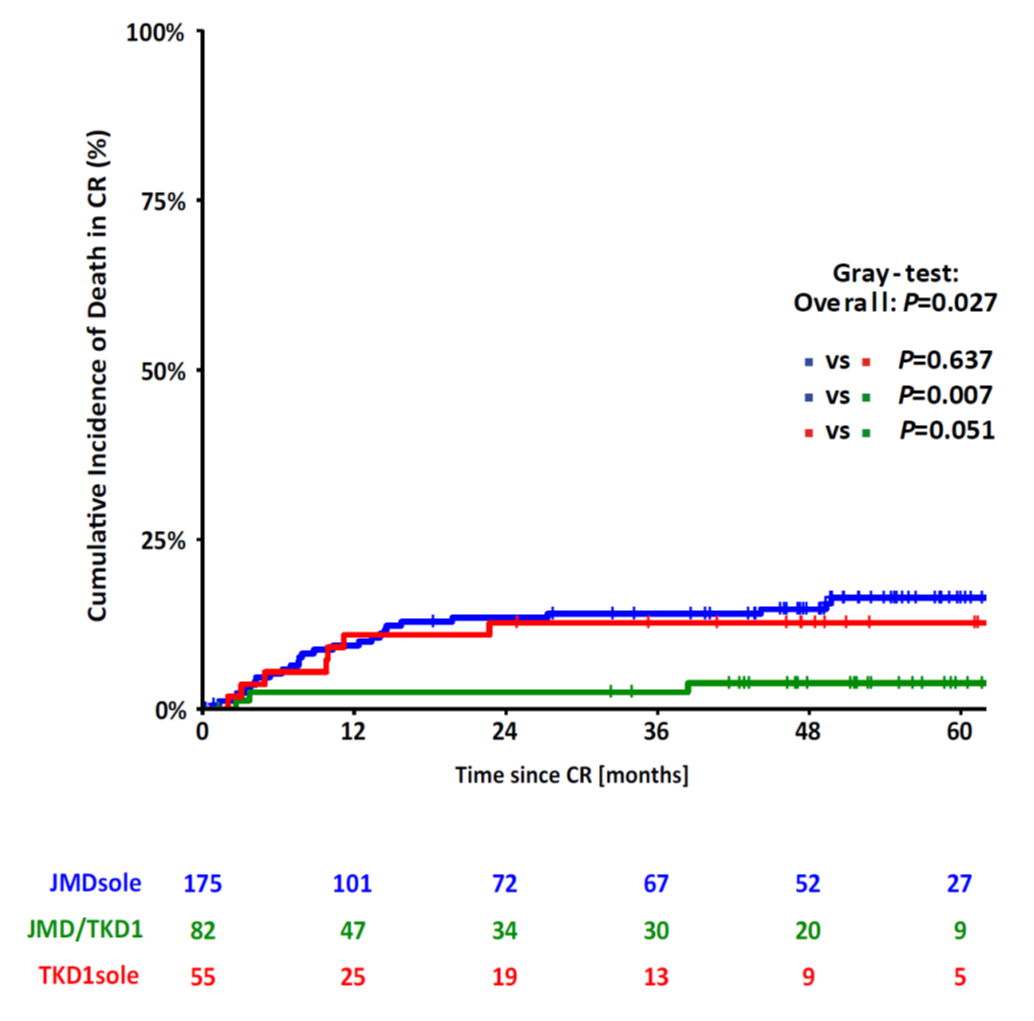


**Supplementary Figure 5.** Overall survival (A) and cumulative incidence of relapse (B) according to ITD IS, *NPM1* mutational status, and treatment (midostaurin vs placebo). The results of the pairwise analyses are given in the lower panel.

**
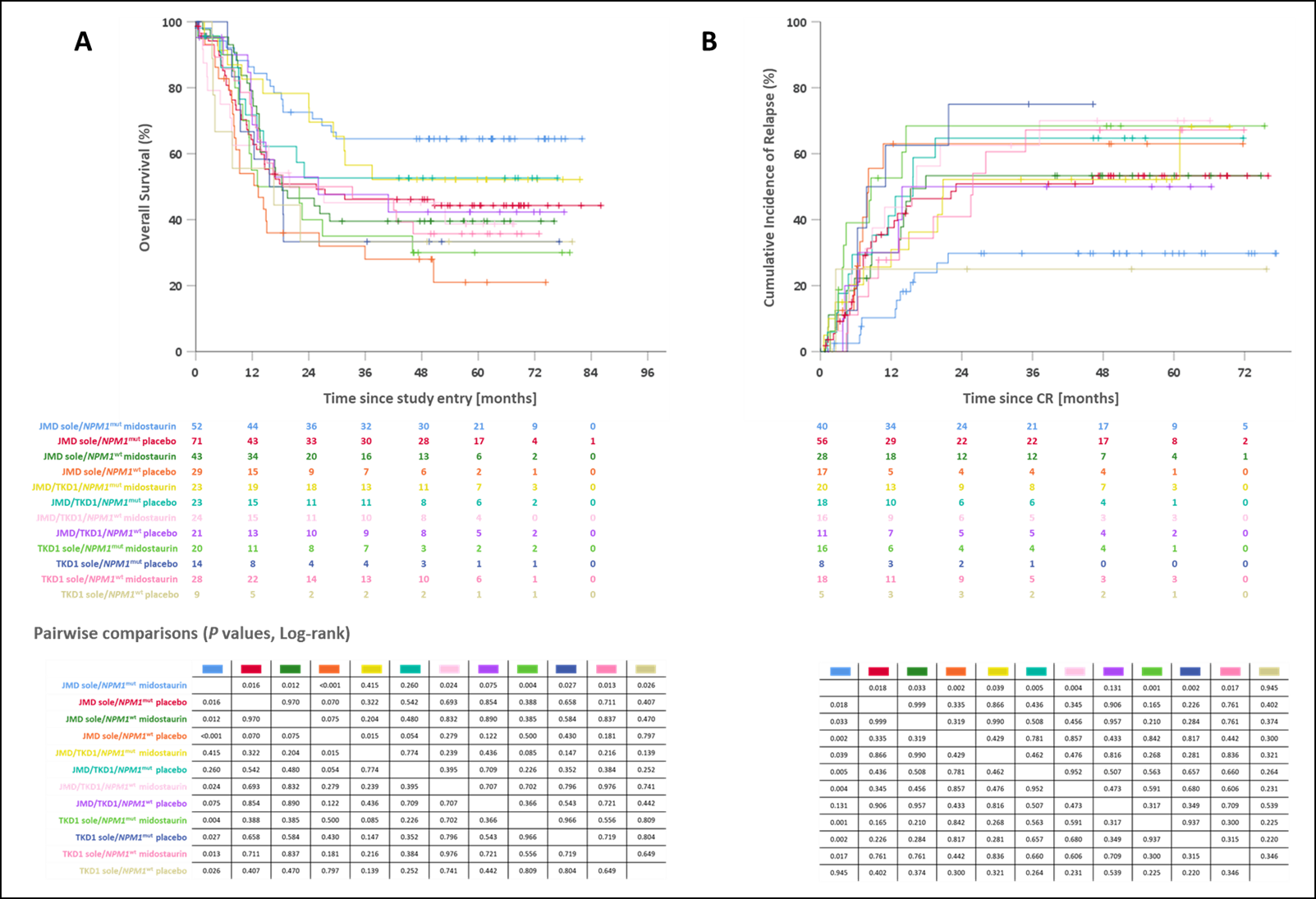
**
